# A Systematic Review of Arts-Based Interventions Delivered to Children and Young People in Nature or Outdoor Spaces: Impact on Nature Connectedness, Health and Wellbeing

**DOI:** 10.3389/fpsyg.2022.858781

**Published:** 2022-03-08

**Authors:** Zoe Moula, Karen Palmer, Nicola Walshe

**Affiliations:** ^1^Institute of Education, University College London, London, United Kingdom; ^2^Faculty of Health, Education, Medicine and Social Care, Anglia Ruskin University, Cambridge, United Kingdom

**Keywords:** systematic review, arts, nature, outdoors, nature connection/connectedness, health and wellbeing

## Abstract

**Background:**

The time that children and young people spend in nature and outdoor spaces has decreased significantly over the past 30 years. This was exacerbated with a further 60% decline post-COVID-19. Research demonstrating that natural environments have a positive impact on health and wellbeing has led to prescription of nature-based health interventions and green prescribing, although evidence for its use is predominantly limited to adults. Growing evidence also shows the impact of arts on all aspects of health and wellbeing. However, what has received scant attention in literature is the interconnection between the two: arts and nature.

**Aims:**

This review synthesizes the literature surrounding the interconnectedness between arts and nature, and their impact on the health and wellbeing of children and young people.

**Methods:**

Eight major electronic databases were systematically searched, while hand-searching included 20 journals, six books, and contact with experts. The review was conducted using the Cochrane handbook for systematic reviews, PRISMA guidelines and TIDieR template. All stages were conducted independently by two researchers and the protocol was published on PROSPERO (Registration no.: CRD42021286574).

**Results:**

Although 9,314 records were identified, only 11 records were included as most studies focused either on arts or nature, but not both. Studies were conducted in United Kingdom, United States, Ireland, Australia, and Hong Kong, in a range of spaces such as forests, woodlands, beaches, parks, fields, gardens, and school playgrounds. The review encompasses data from 602 participants in total.

**Discussion:**

Arts-in-nature offered an inclusive medium to engage all children and young people, especially those who might otherwise remain disinterested about environmental issues and disengaged with educational programs. Further, arts-in-nature provided stimuli to increase nature connectivity, understand environmental issues and explore ways to prevent environmental disasters. This led to higher environmental awareness and pro-environmental behaviors, and potential decrease in eco-anxiety.

**Conclusion:**

Although the quality of qualitative studies was high, the quality of quantitative studies was low or unclear, thus quantitative evidence is still at its infancy. Implications for research, policy, and practice are discussed, such as methods and activities to strengthen future interventions. Scaling-up existing interventions may lead to wider recognition and inclusion of arts-in-nature in future health guidelines, including green prescribing.

## Introduction

In the United Kingdom, one in five children (1.1 million) have reported feeling unhappy with their lives since the beginning of COVID-19 pandemic (Children’s Commissioner, 2021). Clinically significant mental health conditions in childhood have increased by 50%, while two thirds of school children have experienced social isolation and loneliness; also a 50% increase compared to pre-COVID-19 (Children’s Society, 2020). In a recent survey with 2,438 children and young people (Young Minds, 2021), nearly 70% said that the pandemic will have a long-term negative effect on their mental health. In addition to that, the number of children regularly playing in nature and wild places in the last 30 years in the United Kingdom fell by 90% (National Childhood Report, 2012). The People and Nature Survey (Natural England, 2020) suggested that this decline was exacerbated by COVID-19 as six in ten children (60%) reported spending less time outdoors. However, children who reported spending more time outside and more time noticing nature/wildlife were more likely to report that “being in nature makes me very happy” (91 and 94%, respectively, compared to 79% of those who had spent less time). Furthermore, 46% of parents believed their children seem happier outside, and 42% believed that nature is more important than ever.

Increased attention is being placed on the physical environments that children and young people experience due to the continuously expanding wealth of evidence that being in nature and outdoor spaces has positive repercussions on mental health and wellbeing (Nisbet et al., 2009; Bratman et al., 2012; Kamitsis and Francis, 2013). These desirable effects include reductions in stress, anxiety, depression (Mayer et al., 2009; Roe and Aspinall, 2011; Thompson et al., 2012; Park et al., 2013; Beyer et al., 2014; Chawla et al., 2014), whilst simultaneously impacting positively on self-esteem, mood, and confidence (Barton and Pretty, 2010; Roe and Aspinall, 2011; Park et al., 2013; Chawla et al., 2014). This wealth of research has led to prescription of nature-based health interventions, or green prescribing (e.g., Robinson et al., 2020), although evidence for its use is currently predominantly limited to adults.

Benefits for the health and wellbeing of children and young people can be equally obtained through creativity and instances of arts-based interventions. There is significant evidence showing that engagement with the arts can aid physical, cognitive, linguistic, social and emotional development (APPG, 2017), as well as improvements in mental health and social inclusion (Clift and Camic, 2015; Zarobe and Bungay, 2017; Daykin, 2019; Durham Commission on Creativity and Education, 2019). There is also growing research that delves into the impact of arts therapies, including drama, art, music, and dance movement therapy specifically for children and young people (Moula, 2020; Moula et al., 2020). This evidenced impact of creativity and engagement with the arts has been currently applied to guide policy toward supporting wellbeing (Fancourt and Finn, 2019).

Whilst the impact of nature and outdoor environments on health and wellbeing is widely evidenced, likewise, engagement with the arts has been demonstrated to promote health and wellbeing for children and young people. However, what has received scant attention in the literature so far is the interconnection between the two: arts and nature. Therefore, this systematic review aims to synthesize the literature surrounding the interconnectedness between arts and nature, and their effect on the health and wellbeing of children and young people.

To date, information with regards to the theoretical frameworks, techniques, practices, and dosage (i.e., frequency, duration, intensity of sessions) in arts-based interventions delivered in nature and outdoor spaces for children and young people has yet to be synthesized. Further research is required to systematically report on how such interventions have been implemented and to evaluate the quality of existing evidence. The present systematic review aims to address the following research questions:

• What types of arts-based interventions have been implemented in nature and outdoor spaces? What are their theoretical frameworks, techniques, and dosage?
• How do arts-based interventions in nature and outdoor spaces support the health and wellbeing of children and young people?

## Methodology

This systematic review was conducted in accordance with the Cochrane Handbook for Systematic Reviews (Higgins and Green, 2011). Methods were pre-specified and documented in advance in a protocol that was published on PROSPERO database for systematic reviews (Registration no.: CRD42021286574).

The eligibility criteria were determined based on the PICOS framework (Bowling and Ebrahim, 2005) and were independently assessed by two reviewers (KP, ZM) (Table 1). The search strategy included all relevant keywords (Table 2) with regards to (a) arts; (b) nature and outdoor spaces; and (c) children and young people, in four steps:

**TABLE 1:** Eligibility criteria.

	Inclusion criteria	Exclusion criteria
Population	Studies in which the majority (more than 75%) of participants were younger than 18 years’ old	Studies in which the majority (more than 75%) of participants were older than 18 years’ old
Intervention	Interventions or services that meet ALL the below criteria: a. Designed for and delivered to children and young people (aged 0–18) b. Delivered in nature or outdoor spaces (e.g., school playgrounds, parks, green areas) c. Delivered utilizing arts media (e.g., visual arts, music, drama, dance, movement)	Interventions delivered to adults Nature- or outdoor-based interventions that do not include engagement with the arts Arts-based interventions that are not delivered in nature or outdoor spaces Outdoor interventions primarily based on recreational activities (e.g., hiking, camping, air or water sports) but not arts
Outcomes	All outcomes related to health and wellbeing, at the end of the intervention (immediate), up to 1-year post-intervention (=12 months), and more than 1-year post-intervention (>12 months)	Outcomes not related to health and wellbeing
Study design	Quantitative studies: Randomized controlled trials (RCTs), pilot-, cluster-, or quasi-RCTs, quasi-experimental, controlled before and after studies, surveys Qualitative studies: Observational, ethnographic, narrative, phenomenographic, and grounded theory studies Arts-based studies: Children’s artifacts that reveal information regarding their own perspectives Studies with a clear methodology and research question(s)	Reviews, editorials, policy reviews, commentaries, off notes, statements or opinion articles, and studies not published in English Case studies will be included as a table, but will be excluded from the final analysis Studies without clear research aims, question(s), design and methodology

**TABLE 2:** Search terms.

Population Children and young people	Intervention Arts and creativity	Setting Nature and outdoor spaces
Child*	Creativ*	Natur*
Kid*	Art*	Outdoor*
Adolescen*	Music	Outdoor* activit*
Teenager*	Dance	Outdoor* recreation
Youth	Drama	School ground*
Young	Movement	Nature therapy
Pupil*	Paint*	Forest*
Student*	Draw*	Park*
Boy*	Perform*	Green
Girl*		Garden*

The * symbol was used for terms that could have multiple endings.

Step 1: Searching all keywords related to arts combined with OR
Step 2: Searching all keywords related to nature and outdoor spaces combined with OR
Step 3: Searching all keywords related to children and young people combined with OR
Step 4: Combining all the searches above with AND

**FIGURE 1:**
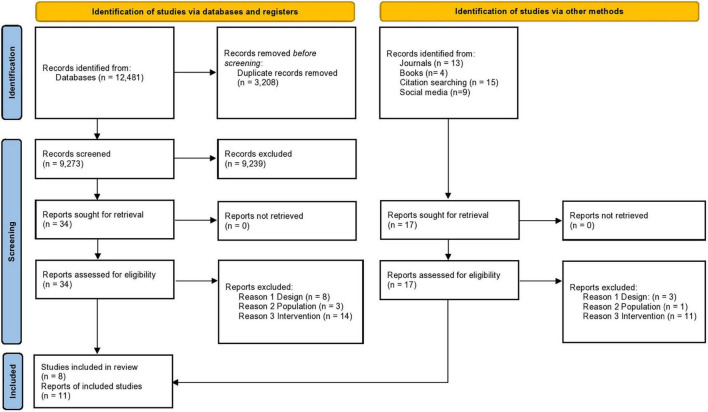
PRISMA flowchart.

Eight major electronic databases were systematically searched, specifically: PsycINFO, CINAHL, ERIC, MEDLINE, DoPHER/ePPI, Education Abstracts Wilson, Campbell Collaboration library and Cochrane library databases, including CDSR, CENTRAL, HTA. The searches also included 20 most relevant journals, six books (Table 3) and contact with experts in this field. Studies were restricted to those published in English until April 30th, 2021. The screening process was recorded in accordance with the Preferred Reporting Items for Systematic Review and Meta-Analysis (PRISMA) guidelines (Page et al., 2020) to ensure that it was undertaken systematically and transparently at all stages (Figure 1). Zotero software was used to identify and remove duplicate titles, and Covidence software was used to organize and manage all relevant information from the studies. The TIDieR Template for Intervention Description and Replication checklist (Hoffmann et al., 2014) was used to extract information based on study and intervention characteristics.

**TABLE 3:** Hand-searching.

Titles of journals searched	No. of papers retrieved	No. of potentially included studies (after title and abstract screening)
Children’s Geographies	144	1
Journal of Adventure Education and Outdoor Learning	382	3
Journal of Outdoor and Environmental Education	327	3
Early Childhood Research Quarterly	134	0
Children and Youth Services Review	92	0
Journal of Childhood	46	0
Child and Adolescent Mental Health	282	0
Early Child Development and Care	310	0
Child Indicators Research	50	0
Children and Schools	19	1
Journal of Early Childhood Research	317	0
Contemporary Issues in Early Childhood	79	0
Early Childhood Research and Practice	27	0
Evidence-Based Child Health	158	0
International Journal of Early Childhood	110	0
Child and Youth Services	361	1
Environmental Education Research	403	0
Children Youth and Environments	371	2
Journal of Outdoor Recreation, Education, and Leadership	190	0
Journal of Environmental Psychology	360	2
**Titles of books searched**		
Environmental arts therapy: The wild frontiers of the heart (Heginworth and Nash, 2020)		
Nature-based expressive arts therapy: Integrating the expressive arts and ecotherapy (Atkins and Snyder, 2017)		
Eco-art therapy in practice (Pike, 2021)		
Adventure therapy, theory, research, and practice (Gash et al., 2020)		
Outdoor and experiential learning: An holistic and creative approach to program design (Martin and Franc, 2004)		
Environmental expressive therapies: Nature-assisted theory and practice (Kopytin and Rugh, 2017)		

Considering that implementing experimental studies with children and young people may be highly challenging for methodological and ethical reasons, qualitative and quasi-experimental designs were also included in this review. As such, our study included experimental and quasi-experimental studies (e.g., RCTs, pilot-, cluster-, quasi-RCTs, controlled before and after studies), qualitative studies (i.e., interviews, focus groups), as well as arts-based studies (e.g., artifacts, photographs, diaries).

The quality of the included studies was assessed by two reviewers (KP, ZM), and any discrepancies were resolved with the involvement of a third reviewer (NW). For the quality analysis of the quantitative synthesis, the assessment criteria according to the ROBIS tool (Higgins and Green, 2011) were based on establishing the following: sequence generation; allocation concealment; blinding of participants; personnel and outcome assessors; incomplete outcome data; selective reporting bias; and other potential risks of bias. For the qualitative synthesis, the following criteria were assessed: (i) credibility: degree of representation of the data from participants’ viewpoints (e.g., cross-checking, peer-debriefing); (ii) transferability: whether findings could be transferrable to other studies or settings (e.g., adequate reporting of demographic characteristics, contextual background); (iii) dependability: whether the methodology was sensible and adequately documented (e.g., peer-review, triangulation, inter-rater agreements); and (iv) confirmability: whether the findings were confirmable through analysis that is grounded in the data (e.g., audit trails) (Hannes, 2011; Higgins and Green, 2011).

With regards to the reviewers’ background, the first reviewer (ZM) has completed her Ph.D. in school-based arts therapies for children’s mental health and wellbeing, and she is currently a postdoctoral research fellow investigating the impact of arts in nature on children’s health, wellbeing, and environmental sustainability. The second reviewer (KP) is a final-year student completing her bachelor’s in education and undertaking this review as part of her research internship. The third reviewer (NW) is a Professor and head of the department of curriculum, pedagogy and assessment at the UCL Institute of Education; her research is predominantly in the fields of geography and environmental and sustainability education.

## Results

As the PRISMA flowchart illustrates, 12,481 records were identified through database searching, which was reduced to 9,273 following duplicate removal through the Zotero software. This large number of records was retrieved due to the wide searching criteria that were adopted to avoid missing potentially relevant articles. However, following title and abstract screening, only 34 records were relevant. Therefore, the wide searching criteria resulted in “noise” and potentially wasted effort; it is unknown if the included studies would have been identified through more narrow searches. Additionally, 41 records were retrieved via other methods, such as hand searching journals and books, citation searching and social media shout-outs.

Eleven reports of studies were included in this systematic review (Bruni et al., 2017; Gray and Birrell, 2015; Gray and Thomson, 2016; Adams and Beauchamp, 2018, 2019, 2020; Murphy, 2018; Arbuthnott and Sutter, 2019; Sobko et al., 2020; Tiplady and Menter, 2021; Moula et al., 2021). However, two studies were reported in more than one article (Gray and Birrell, 2015; Gray and Thomson, 2016; Adams and Beauchamp, 2018, 2019, 2020). Therefore, the findings from eight studies were synthesized.

Table 4 provides a brief description of each study, and Table 5 provides a brief description of each intervention based on the TIDieR Template for Intervention Description and Replication checklist (Hoffmann et al., 2014). Small case studies, although being excluded from the analysis, were included in a table (Table 6) as these could be useful for other reviews of evidence in the future.

**TABLE 4:** Study description.

Author/Year	Journal	Study aims	Study design	Participants	Intervention	Control group	Setting and country	Study outcomes
Adams and Beauchamp (2018, 2019, 2020)	Research Studies in Music Education Int’l Journal of Children’s Spirituality Journal of Outdoor and Environmental Education	To inspire children to feel as experts/artists To investigate changes on children’s behavior and ability to focus when learning	Grounded theory	Sample size: 185 Age: 7–11 years’ old	Music making outdoors	N/A	6 primary schools in rural locations in north and south Wales	Interviews with children and staff: Higher authenticity, autonomy, agency, freedom, confidence, listening skills, emotional engagement and expressiveness Awareness of senses Immersion and improved focus Better use and appreciation of space Experiences of spiritual moments
Arbuthnott and Sutter (2019)	Environmental Education Research	To examine whether songwriting in a natural setting improves nature connectedness, emotional wellbeing and performance on a creative reasoning task	Quasi-experimental—Controlled before-and-after quantitative study	Sample size: 38 Age: 14–15 years’ old Intervention group: 15 Control group: 23	Songwriting outdoors (i.e., national wildlife conservation area)	Songwriting indoors (i.e., school)	High school in North America	Questionnaires: Increased nature connectedness in intervention group, decrease in control Reduction of negative moods in both groups Nominal increase of positive mood in intervention group, no change in control Improvements in creative reasoning but remains unclear whether natural settings enhance this further
Bruni et al. (2017)	Environmental Education Research	To encourage direct connection with nature through activities such as wildlife observations and arts	Quasi-experimental—Uncontrolled before-and-after quantitative study	Sample size: 178 Age: 8–12 years’ old	Multi-faceted program encouraging nature connectedness	N/A	Schools and youth organizations, South California	Computer-based game/test: Increased implicit connectedness with nature
Gray and Birrell (2015) Gray and Thomson (2016)	Journal of Adventure Education and Outdoor Learning LEARNing Landscapes	To delve into the impact of experiential learning and how the arts promote a personal relationship with the environment	Quasi-experimental—Uncontrolled before-and-after multi-methods study	Sample size: 19 Age: 12–14 years’ old Specific characteristics: gifted adolescents	Multi-sensory immersion program outdoors	N/A	High school with gifted and talented students, New South Wales	Questionnaire: Significant improvements in nature connectedness Interviews, observations, artifacts, photo-elicitation: Greater nature awareness, attunement and attachment
Moula et al. (2021)	Child Research Indicators	To explore how the wellbeing of children living in areas of high deprivation can be supported through working with artists in outdoor places	Quasi-experimental—Uncontrolled before-and-after multi-methods study	Sample size: 101Age: 7–10	Eco-capabilities: creative adventuring in nature	N/A	Two primary schools in East Anglia identified as being in areas of poverty and deprivation with minimal cultural provision and infrastructure	Interviews, focus groups, observations, artifacts, diaries: Sense of belonging/existence as part of nature Appreciation of biodiversity Increased resilience and risk-taking Greater collaboration, emotional expression, and empathy Questionnaire: Results not yet reported
Murphy (2018)	Journal of Adventure Education and Outdoor Learning	To determine to what extent the Irish primary school curriculum visual arts construction strand objectives can be achieved through the Forest School approach	Action research	11 children Age: 6–8 years’ old	Delivering visual arts through Forest school approach	N/A	Large, urban, disadvantaged, multi-denominational primary school in Ireland	Observations, videos, photographs and journals: Heightened awareness of nature Focus on the process of artmaking Appreciation for others’ artwork Increased self-esteem Interviews: Parents and staff reported improved independence, organization and social interactions
Sobko et al. (2020)	Nature Research—Scientific Reports	To investigate the impact of the “Play and Grow” intervention on the intestinal microbiome, gut serotonin level, and well-being	Two-arm randomized controlled trial	Sample size: 54 Age: 2–5 years’ old Intervention group: 30 Control group: 24	Play and Grow’ early environmental education program with connectedness to nature component	No intervention	Community centers and kindergartens in Hong Kong	Questionnaires and biomarkers: Significant improvements in connectedness to nature and responsibility towards nature Significant reduction in stress and anger No significant change in microbiota diversity but children with decreased perceived stress had significantly higher gut microbiota richness
Tiplady and Menter (2021)	Journal of Adventure Education and Outdoor Learning	To assess impact on emotional wellbeing and to examine the causal processes involved in the Forest School program	Ethnographic-inspired co-production	Sample size: 16 Age: 5–13 years’ old Specific characteristics: emotional and behavioral difficulties, severe anxiety	Forest School	N/A	School 1: primary Additionally Resourced Centre (ARC) in the UK School 2: secondary specialized school in the UK	Observations, interviews, diaries: Insufficient evidence for wellbeing changes Improved engagement, enjoyment, relationships, independence Internalization of positive self-narratives and sense of achievement

**TABLE 5:** Intervention description (Part 1).

Author/Year	Rationale/Theory/Goals		Type of intervention	Assessment materials		Procedures or strategies	WHO delivered the intervention	HOW it was delivered
Adams and Beauchamp (2018, 2019, 2020)	Outside sounds can be heard more clearly, enhancing children’s focus. It also should allow a creative and aesthetic impact through tuning into the natural soundscape, drawing upon the mind body connection to nature and its range of sensory stimulation. The ambience quietness of these locations should also improve children’s musical ability.		Music -making outdoors	Video-stimulated reflective dialogue in interviews with children Interviews with teachers		Children created music for a ceremonial performance using the setting and its relevant historical context as a stimulus. Animal skin, frame drums, djembes, wooden flutes, and bone flutes were provided. Words were not permitted, but vocal sounds were.	Member of research team, who was an experienced primary teacher and facilitator of outdoor learning	Group sessions with approximately 30 children from each class (6 classes in total)
Arbuthnott and Sutter (2019)	As urbanization increases, urban dwellers must experience more meaningful experiences in natural settings. Music influences emotions, while contact with nature improves emotional wellbeing.		Songwriting for nature	Pre-and-post questionnaires		Self-guided program offering outdoor education songwriting. Camps involved guided hikes, campfire jams, discussions about nature and music, group songwriting, a public show and demo recording.	Three Saskatchewan recording artists	Group sessions with 15 young people
Bruni et al. (2017)	Direct personal experience in nature leads to first-hand knowledge of environmental issues and a land ethic which are necessary to increase environmental caring and responsibility. There is a need to increase environmental awareness through knowledge acquisition and skill-building. Incorporating the environment into the sense of self may also be essential to developing environmental care.		Multi-faceted program encouraging nature connectedness	Computer-based game		Children gathered inspiration from the outdoors, aquariums and natural history museums, and incorporated them into artistic projects (e.g., photographs, paintings, drawings, collages, sculptures). Sessions including visiting nature centers, hikes, nature films, and studying about the environment.	N/R	Group sessions Number of children in each group not reported
Gray and Birrell (2015) Gray and Thomson (2016)	Teaching outdoors in a natural environment promotes an appreciation of and lifelong connectedness to the environment. It is ethically imperative that we allow children to build a relationship with nature and promote long term sustainability.		Multi-sensory immersion program outdoors	Interviews Pre-and-post questionnaires Observations Photo-elicitation and artwork		Eco-pedagogical and arts-based enrichment program. Activities included poetry, writing, drawing, painting, music, performing dance, filmmaking, restoration, and lessons on ecosystems.	Artists, scientists, historians, and Aboriginal elders	Group sessions with 19 adolescents
Moula et al. (2021)	Substantial benefits for wellbeing may be derived from contact with nature, however, children living with high deprivation are significantly less likely to have access to green spaces. They are also less likely to have access to the arts as arts are increasingly marginalized in school curricula. This study explores how children’s wellbeing can be supported through working with artists outdoors.		Creative adventuring in nature	Walking interviews with children Interviews and focus groups with teachers and artists Pre-and-post questionnaires Observations Visual data/artwork		Nature-inspired artmaking, including drawing, sculpturing, song writing, story-making and detailed observations of outdoor surroundings, such as the colors and textures of trees and flowers. Children also reflected on the importance of nature and how to protect the environment.	Artists and teachers working together	Group sessions with approximately 30 children from each class (4 classes in total)
Murphy (2018)	Learning becomes more relevant to the children when situated in real-life scenarios. As a visual arts strand, construction needs a stimulus that nature can provide.		Delivering visual arts, construction strand through Forest School approach	Written reflections by children and teachers Observations Visual data Interviews with staff, parents and children		Children inspired by “land art” artists. Clay creatures created, and children tasked to construct a shelter for theirs	Class teacher, qualified as Forest School Leader	Group sessions with 11 children
Sobko et al. (2020)	Urbanization has reduced children’s ability to interact with nature, a potential cause of depression and stress development in later life. These stresses can be measured by changes in the gut microbiome and fecal serotonin. This program was expected to improve children’s wellbeing, connection to nature, alter their gut microbiome and modulate their fecal serotonin level, reduce aggression and stress levels.		Play and Grow’ early environmental education program with connectedness to nature component	ELISA and 16S rDNA amplicon sequencing to measure fecal serotonin level and gut microbiota profiles Pre- and-post questionnaires		15’ theoretical education (e.g., on nature connectedness) 30’ guided activities with materials found in nature (e.g., creating art with leaves, flowers, stones), practicing awareness of sounds and senses, growing plants, environmental care training	Four research assistants	Group sessions Number children in each group not reported
Tiplady and Menter (2021)	To improve the wellbeing of children and young people currently unable to access mainstream education due to extreme anxiety and/or social, emotional, and behavioral difficulties.		Forest School approach	Observations Student attendance and behavioral incidents Student social and emotional literacy self-assessments Interviews with children and young people, parents/carers, school staff, and Forest School practitioner Creative diaries		Activities included den building, putting up and using hammocks, tree climbing, fire building and cooking, tool use and woodwork, arts and crafts, games and exploration of nature.	Forest School practitioner High levels of teacher support, decreasing across the span of the program	Group sessions One group consisted of 5 children One group consisted of 11 young people

**Intervention description (Part 2).**								

**Author/Year**	**WHERE it was delivered**	**No. of sessions**	**Duration of sessions**	**Intensity/ Frequency of sessions**	**Modifications/ Changes**	**Adherence/ Fidelity assessment**	**Effectiveness assessment**	**Estimation of the effects**

Adams and Beauchamp (2018, 2019, 2020)	Locations with strong prehistoric context (e.g., field containing neolithic chambers) Locations without historical significance (e.g., beach, woodland)	2	4 h	2 consecutive days	N/R	N/R	N/A	Higher authenticity, autonomy, agency, freedom, confidence, listening skills, emotional engagement and expressiveness; awareness of senses immersion and improved focus; better use and appreciation of space; experiences of spiritual moments
Arbuthnott and Sutter (2019)	Camp at the National Wildlife Area, Last Mountain Lake in south-central Saskatchewan, North America	Intervention: 2 Control: 4	Intervention: 2 h Control: 1 h	Intervention: 2 consecutive days Control: 4 consecutive days	N/R	N/R	Nature Relatedness Scale (NRS) Positive and Negative Affect Schedule (PANAS) Short version Depression and Anxiety Stress Scale (DASS-21) Remote Associates Test (RAT)	Increased nature connectedness in intervention group, decrease in the control; reduction of negative moods in both groups; nominal increase of positive mood for intervention group; improvements in creative reasoning
Bruni et al. (2017)	Aquariums, natural history museums, nature and outdoors	No. of sessions varied	Duration varied from several hours to one full day	One month	N/R	N/R	Implicit Association Test for Nature (IAT Nature)	Increased implicit connectedness with nature
Gray and Birrell (2015) Gray and Thomson (2016)	Bundanon Trust’s properties, New South Wales	N/R	N/R	One academic year (4 terms)	N/R	N/R	Nature Relatedness Scale (NRS)	Significant improvements in nature connectedness; greater nature awareness, attunement and attachment
Moula et al. (2021)	School playgrounds, nature resorts, parks	8 sessions	One full day	Once a week	N/R	N/R	Personal Wellbeing Index—School Children (PWI—SC)	Sense of belonging/existence as part of nature; appreciation of biodiversity; increased resilience and risk-taking; collaboration; emotional expression and empathy
Murphy (2018)	School garden	6	N/R	Once a week	N/R	N/R	N/A	Pedagogical objectives of visual arts curriculum strand met Heightened awareness of nature Happiness and contentment in the remainder of the school day
Sobko et al. (2020)	Public parks across Hong Kong	10	45 min	Once a week	N/R	N/R Published protocol: European Nucleotide Archive No. PRJEB34058	Measurement of fecal serotonin level and gut microbiota Connectedness to Nature Scale (CNS) Perceived Stress Scale for Children (PSS-C)	Significant improvements in connectedness to nature and responsibility towards nature; Reduction in stress and anger; No significant change in microbiota diversity but children with decreased perceived stress had significantly higher gut microbiota richness
Tiplady and Menter (2021)	Community garden with a woodland area and a Forest School area.	39	One full school day	Once a week	N/R	N/R	N/A	Insufficient evidence for wellbeing changes;Improved engagement, enjoyment, relationships, independence;Internalization of positive self-narratives

**TABLE 6:** Case studies.

Authors and study title
Bassingthwaighte (2017): Taking dramatherapy into the outside space: The benefits and obstacles when working with children with SEMH issues
Berger (2006): Using contact with nature, creativity and rituals as a therapeutic medium with children with learning difficulties: A case study
Kern and Aldridge (2006): Using embedded music therapy interventions to support outdoor play of young children with autism in an inclusive community-based child care program
Martin (2011): The dramaturgy approach to education in nature: reflections of a decade of International Vacation School Lipnice courses, Czech Republic, 1997–2007
Hunter-Doniger (2020): Seeing the forest through the trees: at the intersection of Forest Kindergartens and art-based environmental education
Pfeifer (2017): Music-Nature-Therapy: Outdoor music therapy and other nature related approaches in music therapy

### Description of Included Studies

This systematic review encompasses data from 602 participants in total, with sample sizes ranging from 11 to 187 participants. Where more than one study had utilized data collected from the same samples (Gray and Birrell, 2015; Gray and Thomson, 2016; Adams and Beauchamp, 2018, 2019, 2020), the participants numbers have been included only once in the systematic review’s total.

Three studies were conducted in the United Kingdom (Adams and Beauchamp, 2018, 2019, 2020; Tiplady and Menter, 2021; Moula et al., 2021), one in Ireland (Murphy, 2018), one in Hong Kong (Sobko et al., 2020), two in the United States (Bruni et al., 2017; Arbuthnott and Sutter, 2019) and one in Australia (Gray and Birrell, 2015; Gray and Thomson, 2016).

All included studies varied in terms of their study design. Studies included action research (Murphy, 2018), grounded theory (Adams and Beauchamp, 2018, 2019, 2020), ethnographic-inspired co-production (Tiplady and Menter, 2021), controlled before-and-after designs (Arbuthnott and Sutter, 2019), uncontrolled before-and-after designs (Bruni et al., 2017; Gray and Birrell, 2015; Gray and Thomson, 2016; Moula et al., 2021), and a two-arm randomized control trial (Sobko et al., 2020). Only two studies made comparisons with control groups. Of these, one control group received no intervention (Sobko et al., 2020) and the other (Arbuthnott and Sutter, 2019) had an active control group which engaged in an indoor, school-based intervention (i.e., indoor song writing). The remaining studies did not utilize control groups.

### Description of Arts-Based Interventions

The type of arts-based interventions delivered in nature and outdoor spaces varied in terms of the structure, content, and activities. The most structured intervention was the Play and Grow early environmental education program utilized by Sobko et al. (2020). It involved 15 min of theoretical education on topics such as nature connectedness, followed by 30 min of guided activities with materials found in nature. For example, children were invited to create art with leaves, flowers, or stones. Other activities included practicing awareness of the sounds in nature, growing plants, and suggestions about how to care and protect the environment. Nature “homework” was also encouraged, such as taking care of own gardens, or growing own plants to maximize the frequency of contact with nature. A detailed description of the intervention structure and content has been published in a separate publication (Sobko et al., 2016).

Three interventions implemented multi-modal arts-based approaches which incorporated drawing, sculpturing, song writing, music-making, story-making, filmmaking, dancing, writing, and poetry (Bruni et al., 2017; Gray and Birrell, 2015; Gray and Thomson, 2016; Moula et al., 2021). Additionally, the “Get to Know” program (Bruni et al., 2017) included visits to nature centers, hikes, watching nature films and studying about the environment. The “Touched By The Earth” program (Gray and Birrell, 2015; Gray and Thomson, 2016) focused on ecosystem restoration, while “Eco-capabilities” (Moula et al., 2021) focused on environmental awareness and sustainability. The charity Cambridge Curiosity and Imagination presents a detailed description of the “Eco-capabilities” sessions (CCI, 2021).

Two interventions focused on outdoor music making (Adams and Beauchamp, 2018, 2019, 2020; Arbuthnott and Sutter, 2019). In the first intervention, children were challenged to create music for a ceremonial performance using musical instruments such as drums, djembes, wooden flutes, and bone flutes. Although vocal sounds were allowed, words were not permitted to facilitate emotional expression through music (Adams and Beauchamp, 2018, 2019, 2020). In the second intervention—Songwriting for Nature—outdoor education song writing workshops were delivered, but the sessions also included hikes, campfires, and conversations around the connectedness between nature and music (Arbuthnott and Sutter, 2019).

Two studies used the Forest School approach (Murphy, 2018; Tiplady and Menter, 2021), which followed the cycle of planning, observation, adaptation, and review in each session. Emphasis was given in the relationship between children and nature, as well as in opportunities for children to take supported risks appropriate to the environment and to themselves. Activities included nature-inspired art and crafts, tree climbing and exploration of nature.

### Description of Duration and Intensity of Interventions

Intervention details surrounding number of sessions were diverse, ranging from two sessions (Adams and Beauchamp, 2018, 2019, 2020) to continuous year-long approaches (Gray and Birrell, 2015; Gray and Thomson, 2016; Tiplady and Menter, 2021). The duration also varied from 45 min (Sobko et al., 2020) to one full day (Tiplady and Menter, 2021; Moula et al., 2021). The frequency and intensity varied from two consecutive days (Adams and Beauchamp, 2018, 2019, 2020; Arbuthnott and Sutter, 2019) to once a week (Murphy, 2018; Sobko et al., 2020; Tiplady and Menter, 2021; Moula et al., 2021). In some cases, the description of duration and intensity were not reported (Bruni et al., 2017; Gray and Birrell, 2015; Gray and Thomson, 2016; Murphy, 2018). Specific details of all interventions are presented in Table 6.

### Settings of Interventions

Most of the studies recruited participants from primary and secondary schools. In terms of primary schools, Adams and Beauchamp (2018, 2019, 2020) collaborated with six schools in rural locations in North and South Wales. Moula et al. (2021) worked with two East Anglian schools identified as being in areas of poverty and deprivation with minimal cultural provision and infrastructure, and Murphy (2018) with an urban, disadvantaged, multi-denominational school in Ireland. In terms of secondary schools, Gray and Birrell (2015) and Gray and Thomson (2016) collaborated with a school with gifted and talented students in New South Wales, while Arbuthnott and Sutter (2019) worked with a high school in North America; further details about the demographics of this school were not provided. Tiplady and Menter (2021) employed both a primary school and a secondary specialized school in the United Kingdom. Bruni et al. (2017) recruited the participants from schools and youth organizations in South Carolina, while Sobko et al. (2020) from kindergartens and community centers in Hong Kong.

All included interventions took place in nature and outdoor spaces. Adams and Beauchamp (2018, 2019, 2020)) utilized rural locations with a strong prehistoric context, such as fields containing neolithic chambers, but also locations without strong prehistoric context, such as beaches and woodlands. Whilst some sites were close to houses and roads, all were situated in quiet areas with no urban noise pollution. Two studies explicitly divulged settings as national outdoor localities. Gray and Birrell (2015) and Gray and Thomson (2016) carried out their intervention at the Bundanon Trust properties in Australia, and Arbuthnott and Sutter (2019) at the National Wildlife Area, which is situated in south-central Saskatchewan in North America. Two studies utilized the school playgrounds and school gardens or nature resorts (Murphy, 2018; Moula et al., 2021). Tiplady and Menter (2021) delivered the sessions in a community garden with an established woodland and a dedicated Forest School area, while Sobko et al. (2020) delivered the sessions exclusively in public parks. Finally, Bruni et al. (2017) delivered their intervention in various locations; some sessions took place in nature, but some sessions were delivered indoors in aquariums and national history museums.

### Theoretical Frameworks

Two studies implemented the Forest School pedagogical approach (Murphy, 2018; Tiplady and Menter, 2021), which was based on the six fundamental Forest School principles (Forest School Association, 2021):

a) long-term process of regular sessions, rather than a one-off or infrequent visits, through the cycle of planning, observation, adaptation and review links between sessions;
b) sessions in woodland or natural environment to support the relationship between the learner and the natural world;
c) variety of learner-centered processes;
d) promotion of resilience, confidence, independence, and creative learning;
e) opportunities to take supported risks appropriate to the environment and to the learners;
f) run by qualified Forest School practitioners.

The former study implemented this approach to teach children the process of art, whilst the latter focused on its application to improve young people’s wellbeing.

The studies conducted by Adams and Beauchamp (2018, 2019, 2020) referred to Waite’s work (2017), who advocated that outdoor learning opportunities are more than simply taking classroom activities outside. In contrast, being outside exposes children in new sounds, such as waves or echoes, which may act both as a valuable resource for musical compositions as well as an emotional stimulus contributing to children’s wellbeing. The authors also referred to work Vygotsky’s (1971) arguing that music can transform our emotional state, and our existential state of being. Another significant concept in this study (Adams and Beauchamp, 2019) was that of spirituality, defined as “an awareness that one is connected to something more, beyond the individual self, but which can be grounded in an existential reality” (de Souza and Watson, 2018, p. 345). Finally, the authors created links to Wilson’s biophilia theory (Kellert and Wilson, 1995), a hypothesis suggesting that humans possess an innate tendency to seek connections with nature and other forms of life, and actualizing this inner tendency leads to both physical and mental health benefits. Sobko et al. (2020) also drew from biophilia theory in their study.

Three studies (Bruni et al., 2017; Gray and Birrell, 2015; Gray and Thomson, 2016; Arbuthnott and Sutter, 2019) drew upon the psychological construct of connectedness with nature (Schultz, 2002; Nisbet et al., 2011) to provide the theoretical framework for their studies. This was defined as the degree to which individuals perceive that they are part of the natural environment (Schultz, 2002). Connectedness with nature involved three key aspects: emotional affiliation to nature, understanding the importance and interconnectedness of all aspects of nature, and seeking regular contact with the natural world (Nisbet et al., 2009). The hypothesis in these studies was that nature connectedness would correlate positively with children’s sense of wellbeing, and the results were therefore analyzed through these lenses.

Finally, Eco-Capabilities (Moula et al., 2021) drew upon Amartya Sen’s work (1993) on human capabilities as a proxy for wellbeing. The term “eco-capabilities” was developed to describe how children define what they feel they need to live a fully human good life through the lenses of environmental sustainability, social justice, and future economic wellbeing (the three pillars of sustainability).

### Outcomes and Outcome Measures

The two most reported outcomes were connectedness to nature and sense of wellbeing, followed by engagement in the learning process.

### Connectedness to Nature

All studies reported a positive impact on children’s and young people’s connectedness to nature, assessed either through quantitative (Bruni et al., 2017; Gray and Birrell, 2015; Gray and Thomson, 2016; Arbuthnott and Sutter, 2019; Sobko et al., 2020), qualitative (Gray and Birrell, 2015; Gray and Thomson, 2016; Adams and Beauchamp, 2018, 2019, 2020; Moula et al., 2021) and arts-based methods (Gray and Birrell, 2015; Gray and Thomson, 2016; Moula et al., 2021).

Although four studies employed quantitative analysis to assess connectedness or relatedness with/to nature, there was still heterogeneity in the outcome measures used. Two studies utilized the Nature Relatedness Scale (NRS; Nisbet et al., 2009; Gray and Birrell, 2015; Arbuthnott and Sutter, 2019). Sobko et al. (2020) used the Connectedness to Nature Scale (CNS; Mayer et al., 2009), while Bruni et al. (2017) implemented the FlexiTwins game version of the Implicit Association Test (IAT Nature; Schultz et al., 2004; Schultz and Tabanico, 2007).

### Sense of Wellbeing

The impact on sense of wellbeing was only measured in one study (Moula et al., 2021) through the Personal Wellbeing Index—School Children (PWI—SC; Tomyn et al., 2013) yet the results have not been reported yet. Sobko et al. (2020), although not assessing changes in the sense of wellbeing directly, evaluated factors that affect (i.e., stress) or are affected by (i.e., frequency of anger) wellbeing. The authors reported significant reductions in children’s stress level, which was assessed through the Perceived Stress Scale for Children (PSS-C; Cohen et al., 1983), and in the frequency of anger, although it was unclear how this was measured.

Similarly, all other studies reported benefits which are associated with children and young people’s mental health and wellbeing. Adams and Beauchamp (2018, 2020) observed enhanced empathetic skills and emotional regulation. Gray and Birrell (2015) noticed improvements in vitality, energy and joy, and Murphy (2018) reported improvements in children’s happiness, self-esteem, and resilience. The improvements in these studies were evaluated through qualitative methods.

Two studies took a more critical stance toward reporting positive outcomes. Arbuthnott and Sutter (2019) reported only nominal increases in young people’s positive mood, while the negative moods were reduced in both control and intervention groups (i.e., singing outdoors compared to singing indoors). Changes in these outcomes were measured through the short version of the Depression and Anxiety Stress Scales (DASS-21; Henry and Crawford, 2005), and the Positive and Negative Affect Schedule (PANAS; Watson et al., 1988), in which nine items from the Elevating Experience Scale (EES; Huta and Ryan, 2010) were added. Tiplady and Menter (2021) observed improved relationships and children’s self-perception through achieving success, which led to higher levels of independence and internalization of positive self-narratives. However, the authors mentioned that the evidence related to wellbeing was insufficient, partially due to small sample (*n* = 16), despite children participating in approximately 39 sessions (1 day per week for a school year).

### Engagement in Learning

Four studies observed increased engagement and immersion in the learning process (Adams and Beauchamp, 2018, 2020; Murphy, 2018; Moula et al., 2021; Tiplady and Menter, 2021). Specifically, Adams and Beauchamp (2018, 2020) noted that being outdoors resulted in children being more calm and focused, while their listening skills were also improved. Moula et al. (2021) observed additional improvements for children with special educational, learning, behavioral needs, as well as for children who had lower self-esteem and less confidence in their academic skills. The sessions were described as empowering and teachers reported significant improvements in children’s confidence, behavior and focus in the classroom (results reported in Walshe, 2021). Engagement in the learning process was assessed through qualitative methods, such as interviews with teachers, children and young people, but standardized measures were not used in any study.

### Other Outcomes and Outcome Measures

This section includes some of the less commonly reported outcomes and outcome measures. These were improvements in children’s sense of authenticity (Adams and Beauchamp, 2018), autonomy and agency (Adams and Beauchamp, 2018; Moula et al., 2021), higher awareness of their senses (Adams and Beauchamp, 2018; Moula et al., 2021), and higher sense of belonging in/existence as part of nature (Moula et al., 2021). Sobko et al. (2020) also observed higher recognition and appreciation of biodiversity. The same authors measured children’s level of fecal serotonin and gut microbiota pre- and post-intervention using the ELISA and 16S rDNA amplicon sequencing (García-López et al., 2014; Marizzoni et al., 2020). No significant changes were found in microbiota diversity, but children with decreased perceived stress were found to have significantly higher gut microbiota richness.

### Quality Appraisal

To contextualize these results, the quality appraisal was crucial as some of these findings should be interpreted with caution. In the quality appraisal table (Table 7) five studies were classified as “Qualitative” and three studies were classified as “Quantitative.” Although two studies adopted a quasi-experimental design (Gray and Birrell, 2015; Gray and Thomson, 2016; Moula et al., 2021), they only had small quantitative elements and for this reason were classified as qualitative.

**TABLE 7:** Quality appraisal.

Qualitative studies				
**Author/Year**	**Credibility**		**Dependability**	**Transferability**	**Confirmability**
Adams and Beauchamp (2018, 2019, 2020)	**High**—Verbatim quotes; member checking; peer debriefing; attention to contradicting cases; independent analysis of data by more than one researcher		**High**—Logical, traceable and clearly documented process; triangulation; reflexivity	**High**—Details regarding reasons for the selection of study participants; demographics; contextual information; setting location; activities	**High**—Details regarding researcher’s background and its impact on study. However, interview questions were not publicly accessible
Gray and Birrell (2015) Gray and Thomson (2016)	**Unclear**—Verbatim quotes but without attention to contradicting cases; unclear whether the analysis was conducted independently by the two researchers		**Unclear**—Logical process but some elements not traceable or clearly documented	**Unclear**—Details regarding participant demographics and activities. However, information regarding intensity and frequency of program not reported.	**Unclear**—Little researcher background provided and its impact on study not discussed; interview questions not publicly available
Moula et al. (2021)	**High**—verbatim quotes; peer debriefing; attention to contradicting cases; independent analysis of data by more than one researcher		**High**—Logical, traceable and clearly documented process; triangulation; peer review; reflexivity	**High**—Details regarding reasons for the selection of study participants; demographics; contextual information; setting location; activities	**High**—Details regarding researcher’s background and its impact on study; Interview questions publicly accessible
Murphy (2018)	**High**—Verbatim quotes; peer debriefing; attention to contradicting cases		**High**—Logical, traceable and clearly documented process; triangulation; peer review; reflexivity	**High**—Details regarding reasons for the selection of study participants; demographics; contextual information; setting location; activities; documentation of modifications	**High**—Details regarding researcher’s background and its impact on study; Interview questions publicly accessible
Tiplady and Menter (2021)	**High**—Verbatim quotes; member checking; peer debriefing; attention to contradicting cases		**High**—Logical, traceable and clearly documented process; triangulation; peer review; reflexivity	**High**—Details regarding reasons for the selection of study participants; demographics; contextual information; setting location; activities	**High**—Details regarding researcher’s background and its impact on study. However, interview questions not publicly accessible

Quantitative studies					

**Author/Year**	**Selection bias**	**Performance bias**	**Detection bias**	**Attrition bias**	**Reporting bias**

Arbuthnott and Sutter (2019)	**High**—No random sequence generation and allocation concealment	**High**—No blinding of participants or personnel	**High**—No blinding of outcome assessments. Assessments were completed during transportation to workshops	**Low**—No attrition reported	**Low**—All outcomes reported sufficiently; acknowledgement of ambiguous results leading to potentially contradictory findings
Bruni et al. (2017)	**High**—No random sequence generation and allocation concealment	**High**—No blinding of participants or personnel	**High**—No blinding of outcome assessments	**Low**—Small attrition rate justified with reasons	**Low**—All outcomes reported sufficiently
Sobko et al. (2020)	**Unclear**—Random sequence generation and allocation concealment claimed but without details	**High**—No blinding of participants or personnel	**Low**—Blinding of outcome assessments	**Low**—Small attrition rate justified with reasons	**Unclear**—All outcomes reported but information about participant demographics missing

Almost all qualitative studies were rated remarkably high in terms of the four quality appraisal criteria: credibility; dependability; transferability; and confirmability. This could be because studies which did not report explicitly the research aims, questions and design (therefore the studies with potentially lower quality) were excluded at the full-text screening stage. Only one qualitative study was rated as “unclear” in terms of its quality (Gray and Birrell, 2015; Gray and Thomson, 2016) because some key details were missing. For example, the duration and frequency of the intervention was not reported, information regarding the researchers’ background was limited, and the interview questions were not publicly available.

All other qualitative studies were rated as high in terms of credibility, for example because verbatim quotes from the participants were used to support the findings, and the results were analyzed independently by more than one researcher. All studies rated high in dependability as they adopted a logical, traceable, and clearly documented research process. Transferability was also rated high because information regarding the reasons for the selection of study participants; demographic characteristics, contextual information, setting location and activities delivered were clearly outlined. Finally, almost all authors reflected on their research background and the impact this might have had on the studies, while interview or focus group questions were publicly accessible, thereby improving the confirmability of the findings.

The quantitative studies were rated significantly lower, partially because most studies were quasi-experimental, therefore methods such as randomization and blinding did not take place. This led to studies being rated as “high” or “unclear” in terms of selection, performance, and detection bias. In the single study that adopted an experimental design (Sobko et al., 2020), information was missing regarding the methods of random sequence generation, allocation concealment, and blinding of the outcome measures. One study also administered the questionnaires during transportation (bus ride) to the workshops and children were expected to complete the questionnaires during that time, increasing the detection bias (Arbuthnott and Sutter, 2019). However, all studies rated low in attrition bias because there were only small attrition rates, and these were reported with justifications. Reporting bias was also rated low in two studies (Bruni et al., 2017; Arbuthnott and Sutter, 2019) but high in Sobko et al. (2020), because necessary information, such as participants’ demographic characteristics, were not reported.

## Discussion

This systematic review gathered evidence from eight interventions involving 602 children and young people. The art-based interventions were delivered in a range of physical environments, such as woodlands, beaches, parks, fields, gardens, and school playgrounds. Their impact is discussed in this section, situated within the existing literature. The implications of this review for future research, policy and practice are also highlighted in this section.

### Cycle of Nature Connectedness

**FIGURE 2:**
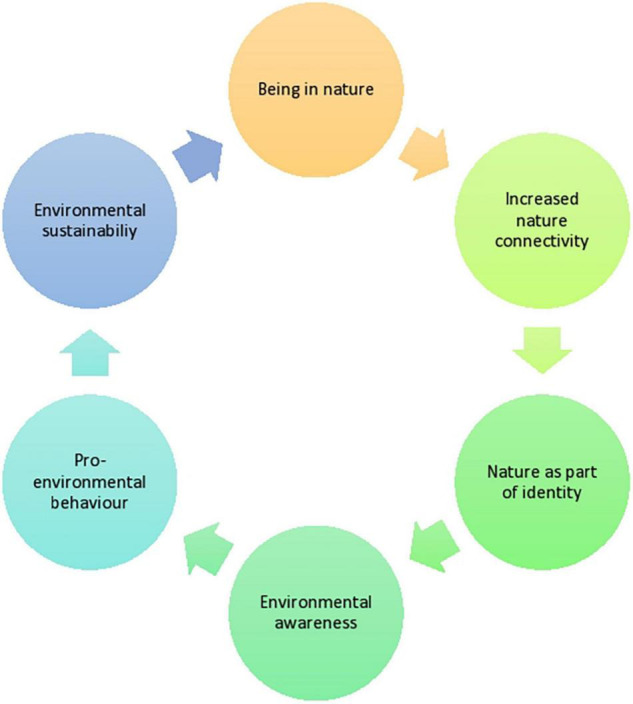
Cycle of nature connectedness benefits.

Nature connectedness was the most commonly reported outcome of interest in this systematic review. Being in nature appeared to lead to children and young people feeling gradually, and increasingly more connected to nature. In turn, feeling connected to nature led to sensing that nature was becoming an important part of their identity—what has been defined as pro-environmental identity (Stets and Biga, 2003)—and came alongside higher awareness of environmental issues. This led to pro-environmental behaviors, behaviors in which children and young people were not only caring for the environment but were also taking protective actions, contributing therefore to environmental sustainability. Finally, this led to higher desire in having sustained contact with nature. Figure 2 aims to illustrate this process as understood through the synthesis of the included studies. However, it is worth mentioning that the process of change appeared to be highly complex, therefore the diagram illustrates only one possible direction of travel out of multiple possible directions.

The findings of this review echo previous studies which have found that exposure to the natural world is a strong predictor of pro-environmental attitudes and ecological behaviors (Mayer and Frantz, 2004; Leary et al., 2008; Nisbet et al., 2009; Frantz and Mayer, 2014). Interestingly, connectedness to nature has been found to be an even stronger predictor of pro-environmental behavior in children than environmental knowledge (Schultz, 2002; Schultz and Tabanico, 2007; Gosling and Williams, 2010; Otto and Pensini, 2017; Barrable and Booth, 2020). Similarly, research suggests that the way that connectedness to nature can be achieved is not through learning in theory about the environment, but by being exposed to the beauty of nature, the emotions that arise while being in nature, and with sustained contact (Ryan et al., 2010; Rainisio et al., 2014; Lumber et al., 2017). Hence, the concept of spirituality that was explored in the Adams and Beauchamp (2019) study may be especially important to better understand the process through which connectedness to nature can be achieved, and how it enhances pro-environmental attitudes (Martin et al., 2020).

More specifically, Adams and Beauchamp (2019) found that outdoor music making provoked a sense of interconnectivity and harmony with nature, leading to experiences of extraordinary, transcendent, or so called “spiritual” moments (Hay and Nye, 2006; de Souza and Watson, 2018; Schein, 2017). During these moments, children expressed that they experienced a sense of entering a heightened reality that included bonding with nature and with each other, and it was described as entering a “new” or a “magic world.” Feelings of joy and peace were also generated during these moments. The authors created links with the Hay and Nye (2006) self-transcendence theory, according to which, it is only when we move beyond egocentricity that we can start to relate genuinely with nature and with each other. These immersive experiences resonated with theories of optimal experiences and “flow” (Csikszentmihalyi, 2002), Buber’s (1937) existential philosophy of dialogue, and Gelter’s (2010) concept of “genuine friluftsliv” (translated as “open air living”)—a commitment to celebrating time outdoors regardless of the weather. Most importantly, these findings echo previous literature arguing that it is possible for spiritual experiences to be achieved in childhood, because they don’t require high cognitive abilities or sophisticated language capacity, but universal human awareness (Hay and Nye, 2006). Arts can be the catalyst to inspire these spiritual experiences, thus reinforcing nature connectedness.

### Interconnectedness Between Arts and Nature

**FIGURE 3:**
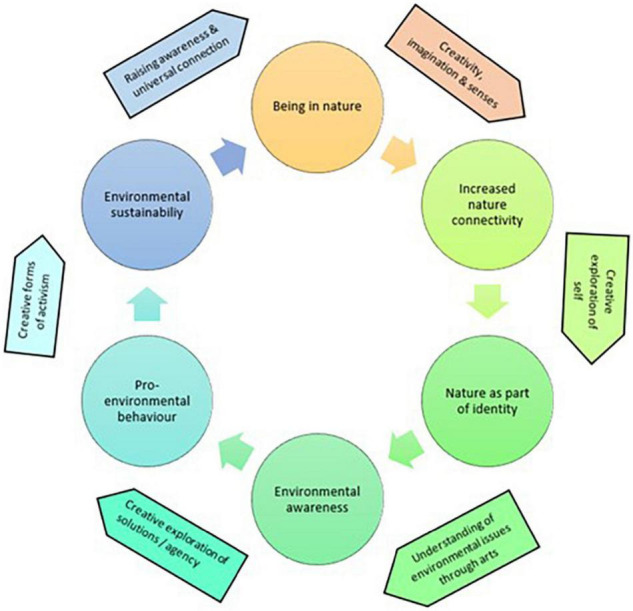
Cycle of arts in nature benefits.

The synthesis of the included studies suggests that arts can offer an inclusive medium to increase nature connectivity, make the relationship with nature explicit, understand environmental issues and explore ways to prevent future environmental disasters, leading to environmental sustainability. Figure 3 aims to illustrate how engagement with the arts were found to provide additional benefits to being in nature and outdoor spaces. However, similar to the previous diagram, the process of change appeared to be complex and can occur via different paths that those illustrated in the diagram.

The included studies suggested that engaging with creativity, imagination and sensual experiences while being in nature can increase nature connectivity. Adams and Beauchamp (2019) reported that one of the first noticeable differences observed was a sensual experience of nature that was achieved through music-making; in this case, the freedom of space outdoors appeared to maximize opportunities for creativity and imagination, while also freeing up the institutional normative behaviors that are expected in the classroom (Waite, 2017). The combination of arts and nature also allowed children and young people to explore the significance of the history of places and the “stories of the land” (Stewart, 2008, p. 82). Through taking part in activities that involved emotional stimulation, self-reflection, and exploration of their identity, children and young people gradually started to perceive themselves as part of the environment, and vice versa, the environment as part of themselves (Bruni et al., 2017; Gray and Birrell, 2015; Gray and Thomson, 2016; Arbuthnott and Sutter, 2019). Furthermore, the outdoor sessions raised awareness of environmental issues through the arts and encouraged the exploration of ideas about what can be done to prevent future environmental disasters from happening, thereby reducing eco-anxiety (Bruni et al., 2017; Gray and Birrell, 2015; Gray and Thomson, 2016; Sobko et al., 2020; Walshe, 2021). Bruni et al. (2017) suggested that arts can engage even the children and young people who might otherwise remain disinterested about environmental issues and/or disengaged with educational programs. Finally, through the arts, children and young people can be involved in creative forms of activism, bringing positive environmental change and environmental sustainability (Inwood et al., 2017). This would encourage more people to spend time outdoors and start their own cycle of nature connectedness.

In summary, and to make this process more explicit, the key stages in the cycle of benefits of the arts in nature, as understood through this review, are as following:

1) Engagement with creativity, imagination and senses can make children and young people spend more time outdoors, increasing connectivity with nature;
2) Identity-focused and self-reflective activities enable children and young people to gradually perceive themselves as part of the environment, and the environment as part of themselves;
3) Activities exploring ideas as to what can be done to prevent future environmental disasters can lead to pro-environmental behaviors and reduce eco-anxiety;
4) Involvement in community-based creative activism can bring wider positive behavioral changes and promote environmental sustainability;
5) Environmental sustainability can encourage, or even enable, other people to spend more time outdoors, starting their own cycle of nature connectedness.

### Impact of Arts in Nature/Outdoors on Sense of Wellbeing

Almost all studies reported improved wellbeing for children and young people, even though most did not explicitly use this term. At least 10 benefits to mental health and wellbeing were reported or observed, including improvements in:

• vitality, energy, and joy (Gray and Birrell, 2015).
• mood (Arbuthnott and Sutter, 2019).
• empathy (Adams and Beauchamp, 2018).
• inner calm and peace (Adams and Beauchamp, 2019; Moula et al., 2021).
• emotional engagement, expressiveness and regulation (Gray and Birrell, 2015; Adams and Beauchamp, 2020; Moula et al., 2021).
• happiness and resilience (Murphy, 2018; Moula et al., 2021).
• stress and anxiety (Sobko et al., 2020).
• relationships and interactions with others (Tiplady and Menter, 2021).
• improved self-perception and internalization of positive self-narratives (Murphy, 2018; Tiplady and Menter, 2021).
• elevating emotions and indicators of eudaimonic wellbeing (Arbuthnott and Sutter, 2019).

Particularly the appreciation of the beauty and the unexpectedness of what can be found outdoors, such as natural sounds and images, appeared to have a positive impact on children’s sense of wellbeing. These findings align with one of the key theories to human-nature relationship, Ulrich’s psycho-evolutionary theory (Ulrich, 1993). Ulrich’s theory posits humans’ innate affiliation with natural environments (Richardson et al., 2018), drawing upon the assumption that natural environments induce positive emotions and feelings. This human-nature connectedness can help children and young people to view themselves as part of a wider ecology which has a positive impact on aspects of wellbeing, such as vitality, creativity, and happiness (Capaldi et al., 2014). Furthermore, connections were made with the PERMA theory of positive psychology (Seligman and Csikszentmihalyi, 2000) which argues that the positive states and traits that children cultivate while in nature (e.g., gratitude, joy, inspiration) contribute significantly to their sense of wellbeing. Seligman and Csikszentmihalyi (2000) have highlighted that the more we enable children and young people to cultivate this state of mind, they happier they can be in the long-term.

The Eco-Capabilities study (CCI, 2021; Moula et al., 2021; Walshe, 2021) developed a novel approach to wellbeing, drawing on Sen’s capabilities work (1993), whereby children were invited to define what is important to them for living a good life through the lenses of environmental sustainability, social justice, and future economic wellbeing. Sen’s human capabilities have been defined as a “a person’s ability to do valuable acts or reach valuable states of being” (Sen, 1993, p. 30). The list of capabilities (Nussbaum, 2000; Biggeri, 2007) include a range of human “functionings” that go beyond the notion of subjective and economic wellbeing (Nussbaum, 2011), aiming to provide opportunities for achieving a state of physical, emotional, intellectual, and existential wellbeing in life (Delors et al., 1996). Among the 101 children who participated in this study, the capabilities that appeared to have been developed the most were the senses and imagination, autonomy, safety, and emotional expression. Based on these, a list of “eco-capabilities” to define children’s wellbeing is currently being developed (Walshe, 2021).

### Implications for Future Research, Policy, and Practice

Although the included qualitative research was rated by both reviewers to have high quality standards, this review suggests that quantitative research is still at its infancy. Only five studies with quantitative elements were identified, of which only three had substantial quantitative elements, and only one was experimental. The quality of the single experimental study was also rated as “low” or “unclear.” Considering the limited funding available for research on this area, national and international collaborative efforts would be of high importance to scale up the existing interventions to larger experimental studies, and to increase the recognition around the value of such interventions especially for policymakers.

The included quantitative studies explored different outcomes, or the same outcomes but using different outcome measures. This led to high heterogeneity in the results, thus a meta-analysis was not feasible. The two most reported outcomes were connectedness to nature and sense of wellbeing. The Nature Relatedness Scale (NRS; Nisbet et al., 2009) was the most commonly used outcome measure, although Gray and Birrell (2015) suggested that it did not explore significant concrete questions about how students felt about nature, and therefore other questionnaires should be considered. There was no outcome measure in common with regards to wellbeing. Future studies should consider the implementation of the most valid, reliable, and widely used outcome measures to allow more accurate comparisons with other studies, replication, as well as future meta-analyses. The Salazar et al. (2020) guide to assessing connection to nature is also highly recommended for decisions regarding outcome measures in future research. Arts-based outcome measures as embedded assessment tools may also be beneficial in capturing changes, particularly for children and young people who might find difficult to verbalize their perspectives (Flowers et al., 2015; Staples et al., 2019).

It would be beneficial for future studies to identify the parameters leading to substantially different effects, such as the duration, frequency, or intensity of the interventions. The sessions in all included studies varied significantly, and there was no evidence to suggest that longer sessions led to better outcomes. In contrast, even as little as two sessions found improvements in nature connectedness and experiences of spirituality (Adams and Beauchamp, 2019), although it is unclear how sustained these benefits might be. In this review, there was no evidence regarding the sustained effects of the included interventions. Although it may be challenging to follow-up children and young people who move to different grades or schools, this would help to understand whether long-lasting changes occurred, or whether the reported effects were due to participants’ temporary confidence that their life could be different following the interventions (Younge et al., 2015). As such, more evidence on the number, duration, and frequency of sessions that could lead to substantial and sustained benefits, could increase the public recognition of such interventions from policymakers and their inclusion in national and international guidelines, such as the National Institute for Health and Care Excellence (NICE) guidelines.

Another important element of the interventions was the space that the sessions were taking people. Adams and Beauchamp (2019) observed that outdoor spaces introduced new and unfamiliar stimuli, such as the sounds of waves or echoes from caves, which provided opportunities for creativity and imagination. However, they emphasized that distractive noise presents threats such as, disruption of attention and behavior, masking of important signals, and spurious physiological stimulation (Hatch and Fristrup, 2009). They found that the sonic environment had the greatest impact on children’s creative and spiritual experiences. Other distractions commonly reported in literature is the lack of cleanliness and amenities, which negatively influence the use of green spaces, even when these are available (Akpinar, 2016; Wood et al., 2018). Therefore, optimizing the conditions of space to make it safe, attractive, and welcoming should be prioritized in future research and policies.

Although it is likely that the cost of the included interventions was high, information regarding the cost to design and deliver the interventions was not available in any study. In the interest of increasing funding opportunities for future research, it is important to focus on the cost-effectiveness of arts in nature and outdoors compared to other interventions (e.g., only arts-based or nature-based interventions). This would also help to identify the additional value and mechanisms of change that occur when brining arts and nature together.

Furthermore, the only experimental study directly investigating physical health outcomes (Sobko et al., 2020) suggested that exposure to bacteria from natural environments could positively correlate to behavioral changes. They recommended future studies to explore the links between gut microbiota, connectedness to nature and psychosocial behaviors.

The impact of working with artists should also be explored in future research. Arbuthnott and Sutter (2019) argued that artists played a crucial role in shifting cultural norms and values, such as normalizing “mistakes,” while Gray and Birrell (2015) observed that the direct contact with professional artists who addressed the students as serious artists maximized the benefits of the sessions and supported the development of children’s identity as artists (Hay, 2019). However, given the high financial cost of the interventions, which is often unaffordable for schools, it would be valuable to investigate whether similar benefits could be observed if the sessions were delivered by teachers. Adams and Beauchamp (2018) highlighted that, if the sessions were to be delivered by teachers, less didactic pedagogies would need to be adopted to give students more opportunities for agency, creativity, and to develop higher self-confidence. It is worth noting that Murphy (2018) observed that teachers often lacked confidence in delivering arts-based sessions, outdoor sessions, or both. Yet this lack of confidence could be due to the time constraints that prevent teachers from investing time and effort in the design and implementation of such sessions (Davies et al., 2014). Therefore, future research should focus on what support teachers need from artists and from their schools to achieve the desired effects. Future policies should also recognize the importance of having dedicated time for outdoor arts-making, embedded within the curriculum, to avoid overwhelming the teaching staff.

Finally, through this review it was possible to identify the impact of specific activities on children and young people. Specifically, starting with simple, familiar and fun activities appeared to increase nature connectivity and make children and young people want to spend more time outdoors. Identity-focused and self-reflective activities led to some children gradually perceiving themselves as part of nature, and nature as part of their identity. Activities raising awareness of environmental issues led to higher pro-environmental attitudes and behaviors, while activities exploring ideas to prevent future environmental disasters appeared to increase agency and decrease eco-anxiety. Developing a manual or protocol of practice based on these activities would enable the design of an intervention that can be piloted for larger experimental studies and replicated with larger populations, such as schools, at a national and international level.

### Strengths and Limitations

This review employed a rigorous methodological design as we conducted extensive searching across multiple databases and we contacted experts in the field to increase our confidence that we have identified the available evidence. We also employed standardized tools, such as the PRISMA guidelines and TIDieR template, to report all stages transparently. The synthesis of the studies in this review have allowed us to develop a framework that illustrates the additional benefits of engagement with the arts in nature and outdoor spaces.

However, it is possible that, although due diligence was taken when carrying out this systematic review, some gray literature, such as research papers that are not formally published in books or journals, may have been missed. Likewise, although a wide range of keywords and search methods was used, it is possible that studies might have been missed if they were inaccurately indexed or categorized in the electronic databases. Considering that the review was limited to English only publications, relevant studies may have been published in other languages.

## Conclusion

Following the analysis of eight interventions involving 602 children and young people, the greatest impact of the interventions was found to be on connectedness to nature and wellbeing. Arts in nature and outdoor spaces provided an inclusive medium to engage all children and young people, especially those who might otherwise remain disinterested about environmental issues and disengaged with educational programs. These findings echoed similar studies suggesting that connectedness to nature cannot be achieved merely through learning in theory about the environment, but by being exposed to the beauty of nature, the emotions that arise while being in nature, and with sustained contact (Ryan et al., 2010; Rainisio et al., 2014; Lumber et al., 2017). Furthermore, the sessions provided creative stimuli to increase nature connectivity, understand environmental issues, and explore ways to prevent environmental disasters. This led to higher environmental awareness and pro-environmental behaviors, as well as a potential decrease in eco-anxiety. Therefore, this review comes in agreement with literature highlighting the importance of children’s and young people’s (re)connection to nature, not only for their wellbeing but also for the sustainability of the planet (Barker, 2007; Louv, 2008; Capaldi et al., 2014; Díaz et al., 2015; Barrable and Booth, 2020).

Although the quality of qualitative studies was high, the quality of quantitative studies was low or unclear, suggesting that quantitative evidence is still at its infancy. Implications and recommendations for future research, policy and practice are discussed to scale up the existing interventions. For example, the methods and activities that could strengthen the impact of future interventions have been identified and synthesized. These could support the development of a protocol or manual to be used in larger experimental studies. There is also a real need for studies to routinely focus on the longevity of the effects identified.

This is the first systematic review to identify, appraise and synthesize the available evidence relating to arts-based interventions delivered to children and young people in nature and outdoor spaces. The implementation of the recommendations for future research, policy, and practice, may lead to the wider recognition and inclusion of arts in nature initiatives in future health guidelines, including through green prescribing.

## Data Availability Statement

The original contributions presented in the study are included in the article/supplementary material, further inquiries can be directed to the corresponding author/s.

## Author Contributions

ZM: first reviewer, conceptualization, design, data collection and analysis, quality appraisal, writing of original draft, and visualization. KP: second reviewer, data collection and analysis, quality appraisal, and review of written report. NW: third reviewer in case of discrepancies, review of written report. All authors contributed to the article and approved the submitted version.

## Conflict of Interest

The authors declare that the research was conducted in the absence of any commercial or financial relationships that could be construed as a potential conflict of interest.

## Publisher’s Note

All claims expressed in this article are solely those of the authors and do not necessarily represent those of their affiliated organizations, or those of the publisher, the editors and the reviewers. Any product that may be evaluated in this article, or claim that may be made by its manufacturer, is not guaranteed or endorsed by the publisher.
